# Dysregulation of Circulating FGF19 and Bile Acids in Primary Biliary Cholangitis-Autoimmune Hepatitis Overlap Syndrome

**DOI:** 10.1155/2020/1934541

**Published:** 2020-06-11

**Authors:** Zhanyi Li, Yu Liu, Fangji Yang, Jiahui Pang, Yuankai Wu, Yutian Chong, Xiangyong Li

**Affiliations:** ^1^Department of Infectious Diseases, Third Affiliated Hospital of Sun Yat-sen University, Guangzhou, Guangdong 510630, China; ^2^Department of General Surgery (Thyroid and Breast), Third Affiliated Hospital of Sun Yat-sen University, Guangzhou, Guangdong 510630, China

## Abstract

**Background:**

Primary biliary cholangitis-autoimmune hepatitis overlap syndrome (PBC-AIH OS), which exhibits features between autoimmune hepatitis and cholestasis, is a common condition and usually shows a progressive course toward cirrhosis and liver failure without adequate treatment. Synthesis of bile acids (BAs) plays an important role in liver injury in cholestasis, and the process is regulated by fibroblast growth factor 19 (FGF19). The overall role of circulating FGF19 in BA synthesis and PBC-AIH OS requires further investigation.

**Methods:**

We analyzed BA synthesis and correlated clinical parameters with serum BAs and FGF19 in 35 patients with PBC-AIH OS. Serum concentrations of 7alpha-hydroxycholest-4-en-3-one (C4) were used to quantify the synthesis of BA directly.

**Results:**

Serum FGF19 levels were higher, while C4 levels were substantially lower in PBC-AIH OS patients than those in healthy controls. Circulating FGF19 levels strongly correlated with C4 (*r* = −0.695, *p* < 0.0001), direct bilirubin (*r* = 0.598, *p* = 0.0001), and total bile acids (*r* = 0.595, *p* = 0.002). Moreover, circulating FGF19 levels strongly correlated with the model for end-stage liver disease score (*r* = 0.574, *p* = 0.0005) and Mayo risk score (*r* = 0.578, *p* = 0.001).

**Conclusions:**

Serum FGF19 is significantly increased in patients with PBC-AIH OS, while BA synthesis is suppressed. Circulating FGF19 primarily controls the regulation of BA synthesis in response to cholestasis and under cholestatic conditions. Therefore, modulation of circulating FGF19 could provide a promising targeted therapy for patients with PBC-AIH OS.

## 1. Introduction

Autoimmune liver diseases (AILD) comprise a spectrum of immune-mediated diseases targeting hepatocytes and bile ducts and involve autoimmune hepatitis (AIH), primary biliary cirrhosis (PBC), and primary sclerosing cholangitis (PSC). A subgroup of patients may exhibit features of 2 different autoimmune liver diseases (commonly defined as overlap syndromes), with PBC-AIH overlap syndrome (PBC-AIH OS) being the most common [1, 2]. The reported morbidity of PBC-AIH OS is 2 to 20% in PBC patients [1, 3]; however, it is generally presumed that the morbidity of PBC-AIH OS is almost 8–10% in adult patients with either AIH or PBC [4, 5]. It is reported that patients with PBC-AIH OS are vulnerable to developing complications related to end-stage liver disease (esophageal varices, gastrointestinal bleeding, ascites, and liver transplant) and lower 5-year survival rates [6, 7].

Within the spectrum of immune-mediated diseases targeting the hepatocytes and bile ducts, PBC-AIH OS patients present with characteristics of both hepatocellular injury (AIH) and cholestatic features (PBC) [1]. Cholestasis is one of the key characteristics of the PBC-AIH OS, and the accumulation of bile acids (BAs) in the liver can play a significant role in consequent progression of the disease, involving inflammation, fibrosis, and ultimately cirrhosis, cancer, and liver failure [8–12]. BA synthesis plays an important role in liver injury in cholestasis.

The control of BA homeostasis occurs through a complex network of pathways regulated by nuclear receptors in the liver [13]. One of the key regulators is fibroblast growth factor 19 (FGF19). As part of a complex enterohepatic feedback mechanism, FGF19 is a hormone that is secreted in the intestine and is transferred to the liver through portal circulation. When FGF19 binds to its receptors, fibroblast growth factor receptor 4 and *β*-Klotho, the mitogen-activated protein kinase pathways are activated and the expression of cytochrome P450 family 7 subfamily A member 1 (CYP7A1, the rate-limiting enzyme for BA synthesis) is suppressed and the synthesis of BA is inhibited [14]. FGF19 is not expressed in normal liver tissue, but its expression is increased under conditions of both extrahepatic and intrahepatic cholestasis [14, 15]. Previous studies suggest that circulating FGF19 are increased in patients with various liver diseases, involving alcoholic hepatitis as well as PBC [16, 17]; however, the general function of serum FGF19 and synthesis of BA in PBC-AIH OS is still unclear and warrants further investigation. These studies could provide implications for potential therapeutic benefits derived from pharmacological suppression of BA synthesis through modulation of CYP7A1 expression and/or activity. In the presented study, we employed the use of measuring serum concentrations of 7alpha-hydroxycholest-4-en-3-one (C4) to directly quantify the synthesis of BA in patients with PBC-AIH OS, where FGF19 and relevant clinical parameters were investigated. Our assessment of the correlations between serum FGF19, synthesis of BA, cholestasis, and prognosis advances our understanding of the pathophysiology of PBC-AIH OS and allows the potential therapeutic benefits of suppressing hepatic BA synthesis to be evaluated.

## 2. Patients and Methods

### 2.1. Patients

All of the enrolled patients were diagnosed and followed up at the Third Affiliated Hospital of Sun Yat-sen University between 2014 and 2018 in this study. The current study was approved by The Hospital Ethics Committee of the Third Affiliated Hospital of Sun Yat-sen University. PBC-AIH OS was diagnosed depending on the Paris criteria, which is commonly employed to diagnose PBC-AIH overlap, requiring at least 2 of the 3 accepted key criteria for the diagnosis of PBC: (1) alkaline phosphatase (ALP) ≥ 2 upper normal limit (UNL) or *γ*-glutamyl transpeptidase (GGT) ≥ 5 ULN, (2) seropositivity for antimitochondrial antibody (AMA), and (3) a liver biopsy specimen presenting florid bile duct injuries. The concomitant diagnosis of AIH requires the existence of at least 2 of the following criteria: (1) alanine aminotransferase (ALT) levels and/or aspartate aminotransferase (AST) ≥ 5 ULN, (2) seropositivity for anti-smooth muscle antibody (ASMA) or serum immunoglobulin G (IgG) levels ≥ 2 ULN, and (3) a liver biopsy presenting moderate or severe periportal or periseptal lymphocytic piecemeal necrosis [4]. Cirrhosis was diagnosed according to either liver biopsy or an image, such as ultrasonography computed tomography, or magnetic resonance imaging. There were 10 individuals with no clinical findings (according to the previous medical records), physical examination, or serum biochemistry which were involved as healthy controls in this research. Blood samples were acquired with the written consent from the patients and approval of the hospital ethics committee. The standard clinical laboratory methods were used to measure the biochemical parameters.

The treatment for PBC-AIH OS patients was based on the clinical guideline and adjusted according to the individual clinical feature of patients. The PBC-AIH OS patients were treated with UDCA at a standard dose. We used immunosuppressive treatment and UDCA in patients with severe interface hepatitis, and consideration in patients with moderate interface hepatitis [18–20]. A biochemical response for the AIH component was designated according to guidelines from the American Association for the Study of Liver Disease [19], while a biochemical response for the PBC component was designated according to the Paris criteria [20]. Patients who met the recommended response criteria for both AIH and PBC were designated as biochemical responders.

### 2.2. Measurement of Serum FGF19 and C4

The levels of serum FGF19 were measured by the sandwich enzyme-linked immunosorbent assay (ELISA) (BioVendor, USA), according to the manufacturer's instructions. The stable isotope dilution and multiple reaction monitoring methods were used to measure serum C4 concentrations; HPLC and ionized by electrospray ionization were used to separated C4 from other sterols [21]. The analytic measurement of this method is range from 0.5 ng/mL to 200 ng/mL.

### 2.3. Statistical Analysis

The baseline demographic and clinical characteristics are listed as percentages or means with standard error of the mean (SEM); Student's *t* or Mann-Whitney *U* tests were employed to estimate the continuous data when chi-square or Fisher's exact tests were employed to estimate the categorical data. Pearson's correlation or the Spearman rank method was employed to estimate the correlation of data. Included assessment of all parameters showing highly significant correlations in the univariate analysis as covariates, multiple linear regression analyses were conducted to identify independent relationships and adjust the effects of covariates. SPSS statistics version 19 (IBM, Armonk, NY, USA) was employed to perform statistical analyses. All analyses were two sided, and differences were defined statistically significant when *p* < 0.05.

## 3. Results

### 3.1. Demographic and Clinical Characteristics of Patients with PBC-AIH OS and Healthy Control Individuals

Thirty-five PBC-AIH OS patients (twenty-one noncirrhotic and fourteen cirrhotic) and ten healthy control individuals were included in this research (Table 1). The serum levels of liver enzymes (ALT, AST, ALP, and GGT), bilirubin (TBIL and DBIL), total bile acids (TBA), globulin (GLB), IgM, and IgG were significantly elevated in patients with PBC-AIH OS (both noncirrhotic and cirrhotic) when compared to healthy control individuals. In contrast, serum albumin (ALB) levels were significantly decreased in patients with PBC-AIH OS regardless of cirrhosis when compared to healthy control individuals. In addition, TBA, INR, Mayo risk score, and MELD score were significantly higher in cirrhotic PBC-AIH OS patients when compared to those in noncirrhotic PBC-AIH OS patients.

### 3.2. The Serum Levels of FGF19 and C4

**Figure 1:**
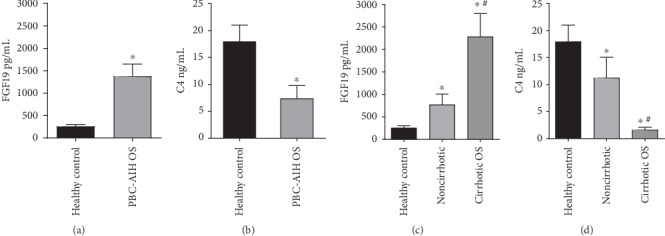
Serum FGF19 and C4 concentrations.

**Figure 2:**
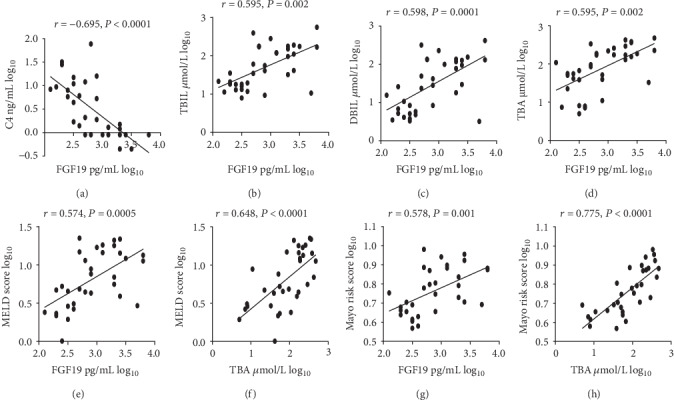
Laboratory and clinical parameters associated with FGF19.

The serum levels of FGF19 were significantly elevated in PBC-AIH OS patients compared to healthy control individuals (1375.41 ± 276.31 vs. 255.94 ± 48.54 pg/mL; *p* = 0.001) (Figure 1(a)). Moreover, the serum levels of FGF19 in noncirrhotic PBC-AIH OS patients were significantly elevated than those in healthy control individuals (771.51 ± 234.97 vs. 255.94 ± 48.54 pg/mL; *p* = 0.028) and the serum levels of FGF19 in cirrhotic PBC-AIH OS patients were significantly elevated compared to those in either healthy control individuals (2281.25 ± 516.32 vs. 255.94 ± 48.54 pg/mL; *p* < 0.001) or noncirrhotic PBC-AIH OS patients (2281.25 ± 516.32 vs. 771.51 ± 234.97 pg/mL; *p* = 0.001) (Table 1) (Figure 1(c)). The serum levels of C4 were significantly decreased in PBC-AIH OS patients compared to those in healthy control individuals (7.39 ± 2.42 vs. 17.98 ± 3.04 ng/mL; *p* < 0.001) (Figure 1(b)). In addition, the serum levels of C4 in noncirrhotic PBC-AIH OS patients were significantly decreased than those in healthy control individuals (11.24 ± 3.82 vs. 17.98 ± 3.04 ng/mL; *p* = 0.015) and the serum levels of C4 in cirrhotic PBC-AIH OS patients were significantly decreased than those in healthy control individuals (1.61 ± 0.51 vs. 17.98 ± 3.04 ng/mL; *p* < 0.001) and noncirrhotic PBC-AIH OS patients (1.61 ± 0.51 vs. 11.24 ± 3.82 ng/mL; *p* = 0.004) (Table 1) (Figure 1(d)). Simultaneously, we detected negative correlations between the serum levels of FGF19 and C4 (*r* = −0.695, *p* < 0.001) (Figure 2(a)).

### 3.3. Clinical and Laboratory Parameters Related to FGF19

Serum levels of TBIL, DBIL, and TBA are typical indicators of cholestasis. Prominently, positive correlations were detected between the serum levels of FGF19 and TBIL (*r* = 0.595, *p* = 0.002), FGF19 and DBIL (*r* = 0.598, *p* = 0.0001), and FGF19 and TBA (*r* = 0.595, *p* = 0.002) (Figure) in patients with PBC-AIH OS (Figures 2(b)–2(d)).

The model for end-stage liver disease (MELD) score (based on a calculation including the international normalized ratio (INR), the bilirubin level, and the creatinine level) has been commonly employed to assess the disease severity and outcomes in liver disease patients [22]. In the present research, a clear positive correlation between the MELD score and the serum levels of FGF19 (*r* = 0.574, *p* = 0.0005) (Figure 2(e)) was detected. Moreover, a clear positive correlation between the MELD score and the serum levels of TBA (*r* = 0.648, *p* < 0.0001) (Figure 2(f)) was detected.

Calculated from a series of potential risk factors (involving the age, bilirubin and albumin levels, prothrombin time, and the existence of peripheral edema and diuretic treatment), the Mayo risk score has been commonly employed to assess the outcomes of patients with PBC [23]. In the present research, a clear positive correlation between the Mayo risk score and serum levels of FGF19 (*r* = 0.578, *p* = 0.001) (Figure 2(g)) was detected. Moreover, a clear positive correlation between the Mayo risk score and serum levels of TBA (*r* = 0.775, *p* < 0.0001) (Figure 2(h)) was detected.

The correlations between serum FGF19 and hepatic inflammation grade and fibrosis stage were evaluated from 13 patients from whose liver biopsy were available. In the present research, we observe a significant positive correlation between serum levels of FGF19 and the patients' fibrosis stage (*r* = 0.581, *p* = 0.037). A positive correlation was detected between serum FGF19 levels and the patients' inflammation grade (*r* = 0.481, *p* = 0.096), but there was no statistical significance.

Univariate regression analysis showed significant positive correlations of FGF19 with bilirubin (TBIL and DBIL), and TBA levels (Table 2). In the present research, we observe a significant positive correlation between serum levels of FGF19 and the patients' fibrosis stage, as well as their MELD score and Mayo risk score (Table 2). A multivariate analysis of these data reveals that TBA was an independent variable of serum FGF19 (*p* = 0.001) in PBC-AIH OS patients (Table 3).

### 3.4. Serum FGF19, Patient Prognosis, and Biochemical Responsiveness

**Figure 3:**
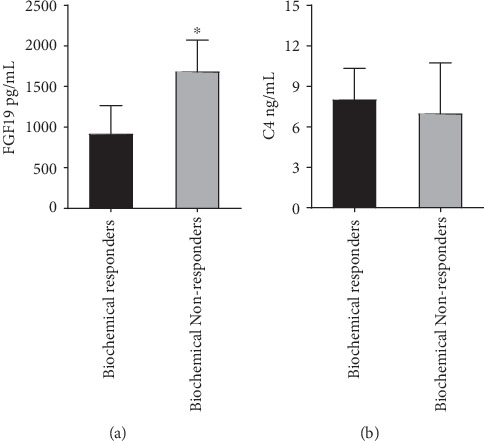
Serum FGF19, C4, and biochemical responsiveness.

Among the 35 patients with PBC-AIH OS, there were 14 biochemical responders and 21 biochemical non-responders. Prominently, serum levels of FGF19 were significantly elevated (*p* = 0.040) in biochemical nonresponders (patients who responded to therapy incompletely) (1681.52 ± 389.65) compared to those in biochemical responders (916.23 ± 348.99) (Figure 3(a)). Conversely, serum C4 concentrations in the biochemical nonresponders were numerically lower, but not statistically different from the levels found in the biochemical responders (6.98 ± 3.76 vs. 8.00 ± 2.33, *p* = 0.143) (Figure 3(b)). As compared to the biochemical responders, serum liver enzyme levels (ALP and AST), bilirubin (TBIL and DBIL), TBA, and Mayo risk scores were significantly elevated in the biochemical nonresponders. On the contrary, serum ALB levels were significantly reduced in the biochemical non-responders. Clinical and laboratory features of patients with PBC-AIH OS are presented in Table 4 as a function of their response to treatment.

In the binary logistic regression analysis, the serum TBA level was a risk factor of nonresponse to treatment in PBC-AIH OS patients (OR 1.028; 95% CI, 1.008 to 1.048; *p* = 0.006) (Table 5).

**Figure 4:**
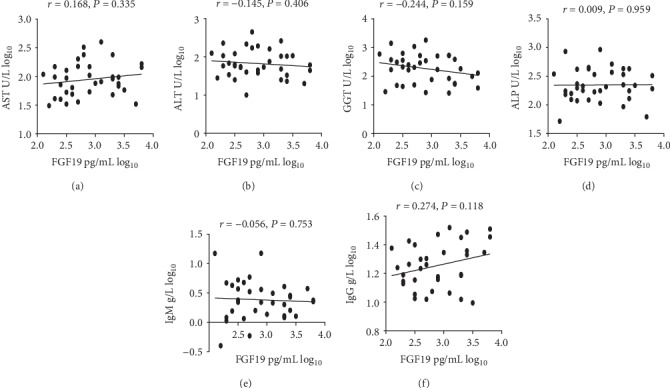
Serum FGF19 levels and liver enzymes, IgG, and IgM.

No correlations between serum FGF19 and liver enzymes (AST, ALT, GGT, and ALP) were observed in patients with PBC-AIH OS. Similarly, no correlations between IgM, IgG, and serum FGF19 (Figure 4) were detected in patients with PBC-AIH OS patients enrolled in the presented research.

## 4. Discussion

The presented research has surveyed the correlation between FGF19, BAs, and other clinical parameters in PBC-AIH OS patients. PBC-AIH OS patients present with features of both hepatocellular injury and cholestasis [1]. PBC-AIH OS patients had significantly increased serum levels of ALT and AST, which are typically employed to indicate the injury of hepatocyte. Similarly, PBC-AIH OS patients had significantly increased serum TBIL, DBIL, and TBA levels, which are typically employed to indicate cholestasis. The role of FGF19 in the regulation of BA synthesis is less well investigated in PBC-AIH OS, given the relatively low incidence. The current report shows for the first time that serum levels of FGF19 are notably elevated in PBC-AIH OS patients, especially patients with cirrhosis. As part of a complicated enterohepatic feedback mechanism, circulating FGF19 regulates synthesis of BA in the liver by inhibiting the expression of the gene encoding CYP7A1, the rate-limiting enzyme for BA synthesis [24]. Serum C4, a direct intermediate product of CYP7A1 in the process of BA synthesis and a marker for BA synthesis [25, 26], was hence significantly decreased in PBC-AIH OS patients, especially cirrhotic patients. The strong correlation between circulating FGF19 and C4, liver fibrosis stage, and the degree of cholestasis (as reflected by TBA and bilirubin) (Figures 2(b)–2(d)) in PBC-AIH OS patients indicates the regulation of BA synthesis (mediated via CYP7A1) is still controlled by circulating FGF19. These data support the hypothesis that the feedback mechanism of physiological FGF19 regulating synthesis of BA is still intact in patients with PBC-AIH OS.

A strong correlation exists between FGF19, and the severity of cholestasis, FGF19, and synthesis of BA (Figure 2) was detected in patients with PBC-AIH OS. On the contrary, serum levels of FGF19 presented no relationship with any of the liver enzymes, IgG, or IgM (Figure 4). These observations are in accordance with previous studies in patients with PBC [17, 27]. One potential explanation is that the level of FGF19 depends mainly on the accumulation of hepatic BAs and the degree of cholestatic condition in PBC-AIH OS patients.

The MELD score is commonly employed to assess disease severity and prognosis in liver disease patients [22], and the Mayo risk score is commonly employed to assess the outcomes of patients with PBC [23]. In the present study, a clear positive correlation was observed between the serum FGF19 levels and the MELD scores (*r* = 0.574, *p* = 0.0005) (Figure 2(e)) as well as Mayo risk scores (*r* = 0.578, *p* = 0.001) (Figure 2(g)). In addition, a strong correlation between the serum FGF19 levels and the liver fibrosis stage was detected in this study. Assessment of the biochemical response indicated that biochemical nonresponders had significantly higher serum levels of FGF19 (Figure 3(a)). In summary, this study suggests that serum levels of FGF19 demonstrate the disease state and could help to indicate the potential prognosis of patients with PBC-AIH OS. Furthermore, a clear positive correlation was observed between the serum TBA levels and the MELD scores (*r* = 0.648, *p* < 0.0001) and Mayo risk scores (*r* = 0.775, *p* < 0.0001). These data support the notion that BA is central to the pathogenesis of cholestasis-induced liver injury regardless of the etiology [28].

In clinical practice, the treatment approach for PBC-AIH OS patients is debated. Therapy recommendations for PBC-AIH OS are empiric, relying on retrospective studies and treatment experience for either PBC or AIH, as randomized controlled trial data are not available for relatively rare diseases. For patients with PBC-AIH OS, the choice between therapy with ursodeoxycholic acid (UDCA) alone or combined with immunosuppressive therapy remains controversial. Several studies have indicated a positive response to the combination of UDCA and immunosuppressive treatment in PBC-AIH OS patients, and this approach is currently the preferred therapeutic regimen [4, 29–31]. However, several studies have also indicated that therapy with UDCA alone is sufficient for PBC-AIH OS patients [32, 33]. Whether the treatment utilizes UDCA alone or in combination with immunosuppressive treatment, we face the problem that the standard immunosuppressive therapy for AIH (steroids with or without azathioprine) [34] could cause an adverse effect on the metabolism of calcium in patients with PBC-AIH OS, leading to osteoporosis [30, 35], whereas UDCA alone appears to be insufficient in controlling hepatic inflammation and may exhibit suboptimal responses in PBC-AIH OS [4].

In this study, we have suggested that BA may be central to the pathogenesis and treatment response of PBC-AIH OS and that serum FGF19 regulates the synthesis of BA in these patients. However, serum levels of FGF19 were significantly elevated (*p* = 0.040) when serum C4 concentrations were lower (however, not statistically different) in biochemical non-responders compared to those in biochemical responders (Figure 3(b)). This result suggests that synthesis of BA was not inhibited sufficiently in patients with PBC-AIH OS who failed to respond to treatment. Targeting these patients for further inhibition of BA synthesis via pharmacological intervention may provide significant therapeutic benefits. In addition, serum levels of TBA were not influenced by the inhibition of BA synthesis in patients with PBC-AIH OS. Collectively, these findings indicate that the physiological FGF19 feedback mechanism regulating the synthesis of BA is not sufficiently robust to counter or reverse the progression of disease, especially in PBC-AIH OS patients who failed to respond to treatment. Activating this mechanism by enhancing resident FGF19, via pharmacological intervention with FGF19 or an appropriate analogue, may supply a powerful and novel therapeutic approach to treating PBC-AIH OS patients. Indeed, NGM282, an analogue of FGF19, has been shown to significantly suppress BA synthesis and reduce ALP, GGT, ALT, AST, IgG, and IgM levels with an acceptable safety profile in PBC patients, including PBC patients unresponsive to UDCA treatment [36, 37]. Treatment with NGM282 was associated with decreases in IgG and IgM levels, indicating a potential immune disease-modifying activity [36]. Though serum levels of FGF19 presented no relationship with any of the liver enzymes or the liver inflammation grade in PBC-AIH OS patients, the BA synthesis suppression and potential immune disease-modifying activity characteristic of NGM282 may present an effective approach to treatment of PBC-AIH OS.

This research has its limitations. First, this investigation was a small-scale single-center cohort study and liver biopsy was available in only 13 patients. Second, it is too limited to obtain a definite conclusion of immunosuppressive treatment effects in PBC-AIH OS patients. Third, the origins of elevated serum FGF19 and the expression of FGF19 in the liver of PBC-AIH OS patients were undefined.

## 5. Conclusions

This study suggests that serum FGF19 levels increase proportionally in response to the degree of cholestasis and severity of the disease in PBC-AIH OS patients. The elevated serum FGF19 lead to inhibition of BA synthesis. Moreover, the measurement of serum FGF19 levels, accompanied by MELD scores and Mayo risk scores, could be potentially employed to demonstrate the severity and prognosis in PBC-AIH OS patients. BA may be central to the pathogenesis of PBC-AIH OS, and FGF19-mediated inhibition of BA synthesis seems to manage in these patients. Pharmacological intervention with an FGF19 analogue (such as NGM282) may provide significant therapeutic benefits in treating PBC-AIH OS patients.

## Figures and Tables

**Table 1:** Clinical and laboratory parameters of PBC-AIH OS patients and healthy controls.

Feature	Healthy controls (*n* = 10)	Noncirrhotic PBC-AIH OS patients (*n* = 21)	Cirrhotic PBC-AIH OS patients (*n* = 14)
Age (years)	53.00 ± 5.07	49.29 ± 2.03	54.57 ± 2.93
Gender (% male)	40	9.52	35.71
FGF19 (pg/mL)	255.94 ± 48.54	771.51 ± 234.97^∗^	2281.25 ± 516.34^∗#^
C4 (ng/mL)	17.98 ± 3.04	11.24 ± 3.82^∗^	1.61 ± 0.51^∗#^
ALT (U/L)	16.30 ± 1.17	196.38 ± 35.31^∗^	170.99 ± 63.54^∗^
AST (U/L)	22.20 ± 1.20	155.67 ± 20.41^∗^	199.33 ± 46.90^∗^
TBIL (*μ*mol/L)	12.29 ± 1.62	75.25 ± 22.56^∗^	135.15 ± 38.36^∗^
DBIL (*μ*mol/L)	3.85 ± 0.46	56.64 ± 18.52^∗^	101.59 ± 28.96^∗^
GGT (U/L)	26.90 ± 5.16	452.71 ± 102.63^∗^	144.36 ± 31.55^∗#^
ALP (U/L)	54.90 ± 4.66	293.48 ± 50.32^∗^	238.14 ± 35.50^∗^
TBA (*μ*mol/L)	2.62 ± 0.48	95.08 ± 21.33^∗^	208.09 ± 40.90^∗#^
ALB (g/L)	44.43 ± 0.66	38.65 ± 0.74^∗^	34.25 ± 1.56^∗#^
GLB (g/L)	27.13 ± 1.16	33.63 ± 1.11^∗^	36.30 ± 3.09^∗^
INR	1.21 ± 0.16	1.06 ± 0.08	1.39 ± 0.13^#^
IgG (g/L)	12.43 ± 0.83	17.00 ± 1.08^∗^	22.25 ± 2.25^∗^
IgM (g/L)	1.19 ± 0.14	4.20 ± 0.89^∗^	1.99 ± 0.25^∗^
Creatinine (*μ*mol/L)	61.22 ± 6.82	65.71 ± 5.35	70.84 ± 12.41
MELD score	n.a.	6.31 ± 1.32	11.72 ± 1.84^#^
Mayo risk score	n.a.	5.56 ± 0.37	6.77 ± 0.44^#^
Liver biopsy available	0	9	4

^∗^ *p* < 0.05 when compared to healthy control. ^#^*p* < 0.05 when compared to noncirrhotic PBC-AIH OS patients.

**Table 2:** Univariable regression analysis of clinical and laboratory parameters associated with FGF19.

Variables	B	SE	*p* value	95% CI	*R* ^2^
TBIL (*μ*mol/L)	0.528	0.126	<0.001	0.271-0.785	0.346
DBIL (*μ*mol/L)	0.423	0.100	<0.001	0.220-0.627	0.352
TBA (*μ*mol/L)	0.479	0.116	<0.001	0.242-0.716	0.339
MELD score	0.724	0.190	0.001	0.336-1.111	0.318
Mayo risk score	2.011	0.608	0.002	0.769-3.253	0.267
Fibrosis stage	0.253	0.107	0.037	0.018-0.487	0.338

**Table 3:** Multivariable regression analysis of clinical and laboratory parameters associated with FGF19.

Variables	B	SE	*p* value	95% CI	*R* ^2^
TBA (*μ*mol/L)	0.470	0.128	0.001	0.208-0.732	0.585

**Table 4:** Clinical and laboratory parameters of biochemical responders and non-responders in PBC-AIH OS patients.

Feature	Biochemical responders (*n* = 14)	Biochemical non-responders (*n* = 21)	*p* value
Age (years)	53.64 ± 2.92	49.91 ± 2.12	0.296
Gender (% male)	14.29	23.81	0.676
Cirrhosis	4	10	0.311
FGF19 (pg/mL)	916.23 ± 348.99	1681.52 ± 389.65	0.040
C4 (ng/mL)	8.00 ± 2.33	6.98 ± 3.76	0.143
ALT (U/L)	163.49 ± 63.69	201.38 ± 34.97	0.105
AST (U/L)	130.26 ± 44.60	201.71 ± 21.06	0.001
TBIL (*μ*mol/L)	34.85 ± 15.83	142.12 ± 29.66	<0.001
DBIL (*μ*mol/L)	20.81 ± 11.84	110.49 ± 22.92	<0.001
GGT (U/L)	269.79 ± 123.46	369.10 ± 77.86	0.089
ALP (U/L)	196.14 ± 57.49	321.48 ± 37.33	0.001
TBA (*μ*mol/L)	40.69 ± 11.49	206.68 ± 28.76	<0.001
ALB (g/L)	40.48 ± 1.11	34.50 ± 0.86	<0.001
GLB (g/L)	35.21 ± 1.80	34.36 ± 2.02	0.772
INR	1.06 ± 0.06	1.28 ± 0.11	0.085
IgG (g/L)	17.85 ± 1.31	20.08 ± 1.81	0.592
IgM (g/L)	3.40 ± 0.98	3.21 ± 0.68	0.710
Creatinine (*μ*mol/L)	85.01 ± 13.79	57.18 ± 2.79	0.069
MELD score	6.32 ± 1.74	9.91 ± 1.50	0.089
Mayo risk score	4.74 ± 0.19	6.78 ± 0.37	<0.001

**Table 5:** Binary logistic regression analysis of clinical and laboratory parameters associated with biochemical response.

Variables	B	SE	OR	*p* value	95% CI
TBA (*μ*mol/L)	0.027	0.010	1.028	0.006	1.008-1.048

## Data Availability

The datasets used and/or analyzed during the current study are available from the corresponding author on reasonable request.
